# Editorial Comment from Dr Kishida to Rare case of a patient with testicular torsion complicated by acute pneumonia, requiring emergency surgery, during the COVID‐19 pandemic

**DOI:** 10.1002/iju5.12418

**Published:** 2022-01-28

**Authors:** Takeshi Kishida

**Affiliations:** ^1^ Department of Urology Kanagawa Cancer Center Hospital Yokohama Japan

In the present case report,^1^ the authors successfully operated a 17‐year‐old boy with testicular torsion complicated by acute pneumonia after 7.5 h from symptom onset. The pneumonia was coincidental, not severe, and usually not prevent minor but emergent surgery like testicular detorsion. However, in the era of COVID‐19, this situation is problematic. To diagnose COVID‐19, the antigen test is soon but less reliable, and PCR test which is more accurate is time consuming. The European Urological association or Japanese Urological association announced guidelines for the Urologic therapy in the COVID‐19 pandemic.^2^, ^3^ They presented that the testicular torsion requires emergent surgery within 24 h to prevent orchiectomy, even though accurate COVID‐19 diagnosis is difficult within that period. In every surgical treatment, special attention will be required to prevent nosocomial infection even after the PCR test as it sometimes has false negative results. In the emergency situation like this case, the caution will be more important.

In the author's Hospital, the special operating room and education for the infectious control were useful in this case. The availability of specific facility is depending on the capacity of health care system and the pandemic situation of COVID‐19. This emergent status may occur in community hospital and we must always ready for COVID‐19 in daily medical practice.

## Conflict of interest

The author declares no conflict of interest.
